# Reproducing and reformulating categories of skill in regional and global migration governance: evidence from India and Bangladesh

**DOI:** 10.1080/01419870.2024.2404485

**Published:** 2024-10-25

**Authors:** Mira Burmeister-Rudolph

**Affiliations:** Department of Political Science, University of Amsterdam, Amsterdam, The Netherlands

**Keywords:** Categorization, migration governance, social construction of skills, South Asia, transnational labor, labor migration‌

## Abstract

Approximately one-third of the world’s 169 million migrant workers come from the Asia and Pacific region, primarily working in temporary, low-wage jobs in the Gulf, where they face human and labor rights issues. In 2003, 12 Asian migrant-origin states formed the Colombo Process to address these labor concerns. This article examines and contrasts two major emigrant-origin states’ – India and Bangladesh –behavior in the Colombo Process, a regional consultative process, and other multilateral migration governance fora, focusing on the social construction of skills. Both countries inherited a colonial legal system of emigration regulation which distinguished emigrants into “high-” and “low-skill” categories, which India continues to reproduce, while Bangladesh aims to reformulate their categorization. This article investigates the how, where, and when of the countries’ emigrant categorization processes by tracing the categories’ origins, their reproduction, and reformulation. This article highlights the significant role migrant-origin states play in the politics of skill.

## Introduction

Since the 1970s, the Gulf Cooperation Council (GCC) countries have been a key destination region for low-wage employed migrants, predominantly from South and Southeast Asia. In these destination countries, they experience major human and working rights abuses (Fernandez 2021). In 2003, twelve Asian migrant-origin states formed the Colombo Process (CP), a regional consultative process (RCP) on labor migration issues “for optimizing the benefits of organized labor migration, whilst protecting their migrants from exploitative practices in recruitment and employment” (IOM n.d.). It operates through senior officials’ meetings, ministerial consultations, whose chairship rotates, and five thematic area working groups (TAWGs). The International Organization for Migration (IOM) functions as the CP’s secretariat (IOM n.d.), with the International Labour Organization (ILO) and United Nations (UN) Women also providing technical and administrative support to two of the thematic working groups.

In migration governance, multilateral fora and RCPs act as sites of norm diffusion and state socialization. Although non-binding and informal, they hold discursive authority: participation is essential for framing issues in global debates (Rother 2019). This paper analyzes and contrasts the categorization of emigrants from two major origin countries – India and Bangladesh – who migrate for low-wage employment and its implications for the countries’ positioning in the CP and other multilateral migration governance fora. Bangladesh has chaired the CP ministerial consultations and TAWGs, whereas India has shown low representation and initiative in the conferences and TAWGs and in taking up chairships.

Active participation in the CP matters as it allows states access to the resources, infrastructure, and technical support provided by IOs and to position themselves as leaders regarding migration governance. India and Bangladesh are two particularly interesting countries to investigate and juxtapose as Bangladesh uses the CP as a platform to proliferate itself as a norm entrepreneur regarding protection and service provision to its emigrant workers and attempts to create a “brand” Bangladesh, whilst India governs its emigrants based on their degree of education and reproduces categories of low- and high-skilled migrants.

India’s current emigration law, the 1983 Emigration Act, retains colonial emigration governance institutions formed by the 1922 Indian Emigration Act. The latter act incorporated previous institutions of emigration control, such as a regulated recruiting system and the Protector of Emigrants (PoE), established by the colonial government in 1864 to monitor indentured migration (Agarwala 2022). India’s present emigration law distinguishes between the Emigration Check Required (ECR) and Emigration Check Not Required (ECNR) categories, whereby ECR-categorized migrants need a so-called emigration clearance. These categorizations are inherently classed: The Indian state assigns the ECR status to persons with a high-school qualification below class 10 intending to migrate to countries of the GCC region. It regards ECR-categorized emigrants as legatees of indentured migrants and similarly as undesirable representatives of India abroad (Natarajan 2022). Emigration from Bangladesh also requires prior registration with the state, albeit for all labor emigrants. Moreover, labor migration is part of the country’s development strategy because of its potential for generating economic and social remittances. Given that India and Bangladesh share a past of colonial migration governance, institutions, and legal systems, this paper asks why and how states’ categorization systems of (e)migrants change or remain?

This paper investigates the how, where, and when of the two countries’ emigrant categorization processes (see Raghuram 2021) by tracing their origin and analyzing their reproduction and reformulation at various sites of migration governance policymaking, using policy documents, official statements, interviews, and secondary literature. It draws on scholarship on the social construction of categories, including categories of skills (Liu-Farrer, Yeoh, and Baas 2021), and investigates migrant-origin countries as “arbitrators of skill” (Liu-Farrer, Yeoh, and Baas 2021, 2240). This perspective emphasizes that the perception of a migrant being “high’” – or “low-skilled” is not an inherent or fixed characteristic but a socially constructed notion and reveals the states’ historical inheritance, interests, and situatedness within the international order embedded in these categories (see Cleton and Meier 2023). My contribution thereby speaks to other work in this Special Issue that emphasizes the relevance of a historically informed analysis in understanding the current use of categories and revealing their more or less evident politicization (see Huhn and Rass 2024).

Building on the understanding that considers international labor migrant governance to unfold in a racialized global political order of nation-states, this research views the countries’ differing involvement in the CP as an outcome of their position within this international order, which incentivizes states with lower political power to introduce new policies to improve their social ranking (Towns 2012) – in this case the professionalization of its workforce. Furthermore, it suggests that the meaning attached to emigrant categories reflects domestic factors, such as the countries’ incorporation of their low-wage emigrants as citizens. This study shows that migrant-origin states are key stakeholders in the field of politics of skill, particularly in the perpetuation and reshaping of skill categories.

This article contributes to the extensive literature on the politics of migration governance in three significant aspects: First, it aims to complement research on the regional institutionalization of migration governance (e.g. Lavenex and Piper 2022). This study’s exploration of RCPs, given the lack of scholarly attention to the CP, enhances the understanding of inter-state consultation mechanisms on migration. These are crucial components of migration governance in South and Southeast Asia, home to one-third of all emigrants worldwide. India and Bangladesh are two particularly relevant countries to study for their total emigrant numbers: India is the largest and Bangladesh is the sixth largest emigrant-origin country globally, and migrants from both countries constitute a considerable population in the GCC countries. Second, while research on the “Postcolonial New World Order” (Sharma 2020) scrutinizes the entanglement of global migration governance with racial logics emanating from colonialism, it often focuses on migration to Europe, Australasia, or North America. To broaden this scope, I explore how the racialized global political order of nation-states mediates states’ positioning and behavior of South Asian countries, with the GCC region as a reference point. Third, this research explores emigrant states’ migrant categorization processes, offering an empirical contribution to previous analyses that have focused mostly on the immigration context (see, e.g. Cleton and Meier 2023).

The first part of this article discusses the conceptual underpinnings of the analysis – how the politics of skills unfold in a racialized system of transnational labor migration and emigrant-origin states draw emigrant policies to position themselves as norm leaders. Next, I outline my research design, including the data collection process and analysis. In the subsequent empirical sections, I first provide an overview of the Colombo Process and the historical formation of emigrant categories in the context of Bangladesh and India, before explaining the two countries’ positioning in the CP and other fields of migration governance by examining underlying factors constituting their construction of skill vis-à-vis their emigrants.

## Conceptual framework

### The racialized international migration regime and the politics of skill

This article builds on an understanding that contemporary transnational labor networks, including between South-, and Southeast Asia and the GCC region, stem from the intersection of historical trajectories of colonialism and racial capitalism (Le Renard and Vora 2021). Fernandez (2021) and Le Renard and Vora (2021) describe the kafala as a system of racialized labor migration governance, which systematically establishes hierarchies among immigrants based on their nationality, class, gender, skill, and migration status. The kafala is a sponsorship system that ties migrant workers to their employers and is practiced across several GCC states and applies to all migrants (Fernandez 2021). Nevertheless, the experience of migrants varies significantly depending on factors like their social and economic capital, encompassing elements such as nationality, gender, social networks, income, and educational degrees (Fernandez 2021). These social classifications also affect their racial capital, the ability to strategically use or downplay one’s appearance, cultural styling, and community ties to gain capital and social advantages (Alloul 2020). Such stratification within the kafala system further perpetuates disparities among the immigrant population, with certain groups enjoying greater access to fair working and living conditions compared to others.

The concept of social construction of skill echoes Iskander’s politics of skill which “becomes a language of power through its ascription of political rights and personhood, and its denial of rights and personhood to those persons represented as unskilled” (Iskander 2021, 10). Like other social classifications, such as class, skill is regarded as a tangible concept, constituted not by competence, but by power. Thereby resulting political implications become obscured. Skill as a classification and a marker of difference is particularly relevant in the context of the GCC countries, but also more generally for labor migration, as it becomes a political category that shapes and stratifies the social and political spaces a person has access to (Iskander 2021). Various actors within specific local, national, transnational, and global settings influence and shape the construction of skill (Liu-Farrer, Yeoh, and Baas 2021).

White-collar workers from the Philippines, South Asian, and Arab countries typically find themselves placed in the middle tiers of this hierarchical structure, while Africans and (South) Asians employed in low-wage jobs occupy the bottom rungs of the ladder (Fernandez 2021). Among the latter, a racialized wage and working conditions hierarchy exists, structured around nationality (see Fernandez 2020; Iskander 2021). Among migrant domestic workers, Filipina women earn the most, followed by Indonesians, Sri Lankans, Bangladeshis, and Nepalis, while Ethiopian and other African women earn the least (Fernandez 2020). Despite performing the same work, Indian construction workers earn a higher income compared to Nepalese, ranked according to racialized interpretations of their physical attributes (Iskander 2021). Consequently, nationalities extend beyond being neutral identifiers of passport ownership; instead, they encompass a system of inherent value ascribed to human bodies, their abilities, characteristics, and social status. In essence, nationalities function as racial categories, implying differentiation within society (Le Renard and Vora 2021) and what constitutes desirable labor.

### Norms in social ranking and emigrant policies as international normative standards

Towns (2012) argues that social hierarchies among states within the international order incentivize states with lower social ranking – countries with lower socio-economic development – to be the first to take up new policies to enhance their social ranking. In establishing the parameters of what is considered normal, norms in the international system not only shape the definition of abnormal and undesirable behavior but also create a basis for ranking states that fail to meet these norms as deficient and inferior (Towns 2012). In the context of migration governance, scholarship has described policies aimed at emigrants and their descendants as being increasingly normal and normative, suggesting that discussions about emigrant policies in origin states are linked to notions of adhering to international norms and democratic standards and representing modernization (Levitt and de la Dehesa 2003).

Multilateral fora act as sites of norm diffusion and state socialization in terms of global migration governance (Rother 2019). They have provided a platform to states, IOs, CSOs, and international policymakers to promote the positively connoted migration-development nexus and a human rights-based approach to migration (Rother 2019). Multilateral fora hold normative authority despite their non-binding and informal nature such as the state-led Global Forum on Migration and Development (GFMD) (Rother 2022). Participation in global dialogues is critical for states to shape the framing of issues. Settings of rotating host state governments have allowed host states to shape the agenda of the forum and to act as norm entrepreneurs and norm leaders (see Rother 2022). States, such as the Philippines and Mexico, have utilized these sites to present themselves as “model states” of diaspora engagement (Gamlen 2014).

Origin states can gain a competitive advantage in the global labor market with their surplus of labor not only by positioning themselves as model states for managing temporary labor migration (Rodriguez 2010; Rother 2022) but also by branding their migrants as “ideal workers” (Chee 2020). The most prominent example is the Philippines, which has successfully gained a reputation as offering best practices of migration management, and “exporting” (Rodriguez 2010) professional, educated, and English-speaking labor, particularly domestic workers (see Chee 2020). Its administrative structures for governing emigration are constitutive of its status as a labor broker state (Rodriguez 2010) which aims at marketing its workers internationally and protecting them abroad (Rother 2022). Part of the “success package” of the Philippines is training before and during deployment through government-licensed centers (Chee 2020). The professional value attributed to Filipino international migrant workers by the global market has allowed the Philippines to introduce minimum wages for its workers in major destination countries (see Chee 2020). Countries, such as Ghana and Indonesia have recognized that pre-departure skills training as a form of up-skilling gives a competitive advantage (see Awumbila et al. 2019; Chee 2020). Consequently, taking an active stance in the CP and additional multilateral migration governance fora does not only provide Bangladesh with symbolic signaling power in terms of being a norm entrepreneur, but being known as adhering to specific training and professional standards also potentially supports the country’s international workers’ positioning in the global labor market.

## Methods and material

This article builds on empirical qualitative data and secondary literature, collected online, in India and the Netherlands from March 2019 to April 2023. The empirical data includes policy documents, summaries, and participant lists of the CP meetings, and six interviews. I held three interviews with CSO stakeholders, two participated in the CP as civil society observers and one in the Thematic Working Group led by Bangladesh; two interviews with representatives of IOs involved in the RCP; and one interview with a Bangladeshi diplomat.^1^

The study relies on tracing Bangladesh’s and India’s understanding of their social positioning and the meaning attached to the category “low-skill” through a close reading of official policy documents, digital information available on the CP and relevant ministries’ websites. I applied an abductive research strategy, which focuses on the iterative process of data collection and data analysis and uses deductive and inductive approaches simultaneously (see Timmermans and Tavory 2012), meaning I revisited all texts at least twice. The abductive approach informs the data collection and analysis in two ways: (1) The empirical focus and research question arose from the surprising observation that, despite similar colonial emigration histories and high migration to the GCC region, India and Bangladesh display different emigration policies and representations of their emigrants in international multilateral fora. (2) I started the data analysis after the first round of data collection, developing concepts, such as emigrant policies as an international normative standard, both from the empirical data and theoretical frameworks.

I also draw on category analysis (see Yanow 2000) and applied Yanow’s (2000, 51) questions of category analysis to analyze the text, to understand how political stakeholders categorize individuals in the context of policy formation. This method deconstructs categories by analyzing their structures and the practices of category-making, to name the unspoken, common sense social meanings underlying policies and their implementation (Yanow 2000). It aligns with the study’s interest in “non-categories” and absences within categories, as it aims to identify “which aspects of the things being so ordered are highlighted by the category schema and which obscured” (Yanow and van der Haar 2013, 232).

## The Colombo process

### Overview, objectives, and operationalization

The primary focus of the Colombo Process lies in labor mobility from Asia to the GCC region. Collectively, the member states of the CP constitute a highly significant source region for global migrant labor: in 2019, approximately one-third of the world’s 169 million migrant workers originated from the Asia and Pacific region (ILO 2023). The CP describes its overarching goals as (1) protecting migrant workers and providing services to temporary overseas contractual workers, (2) optimizing benefits of organized labor migration, and (3) capacity building, data collection, and inter-state cooperation (Colombo Process 2003b).

The CP was created as a platform for origin countries to discuss labor migration and share best practices. It involves consultations to address issues of migrant workers, countries of origin and destination, to offer solutions to protect the welfare of vulnerable workers and to seek constructive dialogue with destination countries. The CP reviews and monitors ministerial recommendations and suggests additional actions (IOM 2023). It employs a dual approach, combining dialogue-based exchanges with programmatic activities, including the provision of relevant technical assistance. It addresses five key thematic priority areas. Each of these areas has thematic area working groups (TAWGs), led by member states that volunteered based on their interest or expertise in the subject matter (IOM 2023).

The CP consists of various meeting formats, including ministerial consultations and senior officials’ meetings (see IOM 2023). Since 2011, CSOs and so-called observers – states of destination regions and relevant IOs – have been invited to take part in the open sessions of the ministerial consultations. Moreover, a rotating chairship system was introduced (Colombo Process 2011). The CP chairship has been held twice by Sri Lanka (2003–2004 and 2013–2017) and once by the Philippines (2004–2005), Indonesia (2005–2009), Bangladesh (2009–2013), Nepal (2017–2021), Afghanistan (2021–2024) (IOM 2023) and India since 2024 (MEA 2024a).

### Competition and common ground

The GCC countries have been key destination regions for migrants from the CP member states. Due to the clustering of target markets in the same region and specific occupational sectors, migrant-origin countries compete to gain increased entry into destination labor markets and improve job placement prospects for their citizens. However, member states share challenges, which has incentivized them to participate in the CP: most migrants from CP member states work in low-wage employment in the GCC region, mostly in the construction, hospitality, and agricultural sectors, or as domestic workers, often facing unpaid wages, dangerous working conditions, overcrowded accommodations, and identity document confiscation (Fernandez 2021). Migrants can significantly increase their income by working abroad due to wage differences between low- and high-income countries like India, Bangladesh, and the GCC countries. However, high emigration costs, including visa fees, travel expenses, and recruitment agency payments, can cause (initial) indebtedness and financial exploitation, resulting in circular migration and permanent temporariness (Piper and Withers 2018).

RCPs, such as the CP, provide (origin) states with the opportunity to informally come together, centering on cooperative dialogue with an emphasis on sharing information and technical cooperation (Von Koppenfels 2001). Since RCPs are non-binding and informal in nature and involve lower commitment costs, states may be more likely to engage in these processes (Von Koppenfels 2001). This is particularly valid for countries that compete for labor markets, and a region with strong inter-state rivalries (e.g. India-Pakistan and India-China) and overall little political and economic integration, such as the South Asian region (see Bishwakarma and Hu 2022). The South Asian Association for Regional Cooperation has not addressed migration as a major issue, in contrast to the Association of Southeast Asian Nations, which issued the Declaration on the Promotion and Protection of Migrant Workers (Wickramasekara 2012).

## The colonial migration governance apparatus in British India and (dis)continuities of categories

The Indian subcontinent had a common system for regulating emigration until 1947. Following the formal abolition of slavery in 1834, the British colonial state instituted measures to control emigration as indentured workers from the British Empire were used to replace enslaved persons in plantation economies. These practices were intended to ensure that indentured workers emigrated voluntarily to distinguish the system from the forced trade of enslaved persons (Mongia 2018). The British colonial authority regulated the emigration of South Asian indentured workers through “coolie agreements” (Singha 2013) and emigration and immigration controls (Mongia 2018) instead of issuing passports, which were selectively given only to educated and wealthy Indians (Singha 2013).

The colonial government established a regulated recruiting system and PoE office in 1864 to monitor these efforts (Rajan, Varghese, and Jayakumar 2010). In 1917, the British colonial administration banned indentured labor migration and introduced a uniform passport for British citizens, covering all Indian emigrants. In 1922, they enacted a new Emigration Act (Agarwala 2022), which retained the PoE institution and introduced a system of emigration clearance for Indians considered to be poor, that is with less than twelve years of schooling.^2^ The act effectively created two distinct emigrant categories and classified its emigrants based on their level of education, thereby implicitly establishing a class distinction, as education functions as a proxy for class in this case (Agarwala 2022).

In 1947 the Indian subcontinent was partitioned into India and Pakistan – the latter consisting of West Pakistan and East Pakistan. In independent India, the 1922 Emigration Act remained in force until early 1983, imposing stringent limitations on Indians emigrating for low-wage employment by issuing only a minimal number of emigration clearances (Agarwala 2022). As mentioned in the introduction, with the subsequent 1983 Emigration Act the Indian state still retained the colonial emigration infrastructure, where potential ECR migrants must obtain a migration clearance from the PoE, effectively regulating and controlling emigration based on classed criteria. This differentiation in emigration policy has had a profound and lasting impact on all subsequent policies related to India’s governance of its emigrants (see Burmeister-Rudolph 2023b).

Reforming the 1983 Emigration Act has been debated for over two decades (Agarwala 2022). The latest attempt, the Emigration Bill 2021, was planned for introduction by the Indian Ministry of External Affairs (MEA) (Kuttappan 2023). Since the announcement, the MEA has not publicly discussed the draft bill’s status, suggesting a halt in proceedings (MEA 2021b). Nevertheless, as Bialas, Lukate and Vertovec (2024) point out, moments of contestation make categories that have been normalized again more visible: The public discussion around the draft Emigration Bill 2021 widely criticized the latter for keeping the ECR/ECNR distinction, but also for intending to facilitate labor emigration rather than to strengthen a rights framework (Bhargava 2022) and proposing to revoke passports or charge fees to those who do not emigrate following the terms of the act (The Draft Emigration Bill 2021, 31(1), 31(2)).

East Pakistan, which became Bangladesh in 1971, fell under the emigration regulations of Pakistan. Pakistan, like India, retained the colonial emigration infrastructure. Bangladesh introduced a new framework with its Emigration Ordinance in 1982, partially repealing but also re-enacting the 1922 Emigration Act.

Similar to the PoE, Bangladesh established the position of the Registrar of Emigrants, with whom emigrants had to register, but this regulation applied to *every* Bangladeshi citizen (see The Emigration Ordinance 1982). The 1982 Emigration Ordinance was repealed by the 2013 Overseas Employment and Migrants Act (OEMA). The OEMA Act is not limited to the regulation of emigrants’ exit but also lays out a legal framework governing recruitment practices and migrants’ rights. It stipulates that all Bangladeshi citizens who emigrate for employment need to register with and thereby obtain a migration clearance from the Bureau of Manpower, Employment, and Training or a Bangladeshi foreign mission (OMEA 2013, 5 (a-g)). Importantly, it promotes the “[a]pplication of the principle of equality” (OMEA 2013, 6). It states that,

[T]he principle of equality is to be applied at all times for overseas employment and return of migrant workers and while providing services or performing any other action under this Act, and no one shall be discriminated on one or more grounds, including, gender, language, birth, colour, age, ethnicity or national origin, political views, religion, ideology, familial, marital or social identity, or regional affiliation, or any other reasons. (OMEA 2013)

In consequence, compared to India, Bangladesh does not only not differentiate between emigrants along social classifications, but explicitly frames its emigration governance framework to be inclusive and non-discriminatory. In the following sections, this article analyzes why and how India continues to govern its emigrants along categories of low- and high-skilled, whereas Bangladesh rejects such differentiation and aims to change the categorization of its migrants towards a “brand” Bangladesh. The analysis thereby focuses on how skill categorizations, in India’s case, are institutionally, legally, and discursively reproduced or, as for Bangladesh, reformulated through re-valuing labor which is categorized as low-skilled. I first outline how state categories play out in the setting of the CP to then scrutinize other policy fields related to migration, such as the social rights of emigrants, development policies, and international conventions, and thereby show the “red threat” of India’s and Bangladesh’s emigrant governance concerning skill categorization processes.

## Bangladesh and India’s positioning in the Colombo Process and multilevel migration governance fora

### Bangladesh

According to official numbers, more than 7.4 million (UN DESA 2021) Bangladeshi citizens are staying abroad – approximately 4.3 per cent of Bangladesh’s total population. Around 3.5 million emigrants live in the GCC region, while Malaysia, Singapore, Jordan, and Lebanon also have been significant destination countries (KNOMAD 2021). Estimates state that around 50 per cent of all Bangladeshi emigrants in the GCC region are labeled as less skilled and 16 per cent as semi-skilled (Agunias, Aghazarm, and Battistella 2015).

Bangladesh has shown active involvement in the CP. It chaired the CP from 2009 until 2013 – when the chairship was still taken up voluntarily (see Colombo Process 2011b) – and hosted the ministerial consultations in 2011 (see Colombo Process 2011c). Moreover, the country leads the TAWG on “fostering ethical recruitment practices” (Colombo Process 2018). Bangladesh further showed initiative by participating in an IOM study (IOM 2015), which analyzed recruitment monitoring mechanisms, compiled good practices of recruitment monitoring and migrant welfare assistance in origin, transit, and destination countries; the ILO study “Comprehensive Mapping and Assessment of Reintegration Measures in South Asian Colombo Process Member States” (Weeraratne, Weerasekera, and Bandara 2022); and in 2020, an ILO pilot survey on migration costs (see ILO Bangladesh n.d.).

As described earlier, Bangladesh’s emigration system does not separate its emigrants into “high” and “low-skill” categories. Instead of reproducing the categorization of Bangladeshi emigrants as “low-skilled”, the Bangladeshi state aims to reformulate this ascription by appraising the contributions of emigrants and therefore re-valuing them domestically. Migration, including into low-wage occupations, is viewed as desirable, not as a stigma, and as a crucial factor in the country’s continuing economic growth. However, Bangladesh cannot unilaterally challenge definitions of skill categories itself. As argued by Ribeiro (2018), the recognition of migrants as skilled professionals is contingent upon negotiations among multiple actors within a network of power. Bangladesh, therefore, demonstrates an appeal to the normative dimensions of migration governance as promoted by the international community, including leadership in international fora, adherence to international conventions, and a national migration governance framework. Additionally, the country practically intends to improve the skill level of its migrant workers and find new labor markets. As I will show in the next paragraphs, Bangladesh acts as a norm entrepreneur to “re-brand” Bangladeshi emigrants internationally.

The importance Bangladesh gives to establishing itself as a leader in migration governance is not surprising given its emphasis on its international migrants as key contributors to the development of the country. Both the seventh (2016-2020) and the eighth 5-year plan (2020-2025) prominently feature low-wage migrants’ placement in international labor markets, and remittances as part of Bangladesh’s development and employment strategy. The seventh 5-year plan’s “Migration for Development” section addresses, among other points, skills, and overseas labor market development; protection, human development, and migration; and South-South cooperation frameworks for migration and development (Government of the People’s Republic of Bangladesh 2015). The eighth 5-year plan acknowledges that “Bangladesh has benefitted tremendously from its large pool of legally [*sic*] migrant labor, especially to the Middle East, both in terms of job creation and huge inflow of remittance income that now accounts for over 5% of GDP”. (Government of the People’s Republic of Bangladesh 2020, 709). It proposes the promotion of overseas job creation and remittances as one of the main pillars of its poverty reduction strategy (see Government of the People’s Republic of Bangladesh 2020). In 2018, Bangladesh presented its “National Strategy and Action Plan on Migration and Development” to operationalize the policy goals outlined in the seventh 5-year plan and the 2016 Expatriate Welfare and Overseas Employment Policy (EWOEP) (Barkat et al. 2017).

The migration policy strategy of the National Strategy and Action Plan and the 5-year plans show a strong overlap with the agenda of the CP, particularly with the areas of the TAWGs. Active participation in the CP serves Bangladesh in achieving its policy goals in several ways. It can make use of the resources, infrastructure, and technical support provided by IOM, ILO, and UN Women. Moreover, Bangladesh does not have much weight and negotiation power in the international system per se, particularly when it comes to migration diplomacy, which is a good reason to come together with like-minded states to increase the bargaining power vis-à-vis destination countries.

Bangladesh establishes its role as a leader and norm entrepreneur in governing international migration discursively, as these following examples show: In addressing the delegation of the GFMD, Bangladesh’s State Minister for Foreign Affairs emphasized “the role that Bangladesh had played in including the concept of a Global Compact in the New York Declaration” (Alam 2022). In the report on Bangladesh’s migration governance framework, Bangladesh’s Foreign Secretary Shahidul Haque writes:

Bangladesh has contributed significantly to the migration discourse, particularly by advocating the inclusion of migration in the Sustainable Development Goals (SDGs). In addition, the Honourable Prime Minister of Bangladesh Sheikh Hasina articulated the idea of the Global Compact for Safe, Orderly and Regular Migration in her address to the United Nations General Assembly in 2016. In addition, Bangladesh is one of the few countries to have mainstreamed migration into the national planning process. (Barkat, Osman, and Ahmed 2020, iii)

Bangladesh’s efforts in migration governance are not limited to the regional level but also encompass global fora and institutions, where it aims to present itself as an important authority. In 2016, it chaired the consultative GFMD, which similarly to the CP is an informal, non-binding, and state-led framework to foster open dialogue on migration and development (see Haque et al. n.d.). Bangladesh is part of the Global Compact for Migration (GMC) Champion Countries Initiative, which is a voluntary piloting process promoting the implementation of the GCM.^3^ In 2019, Bangladesh’s Foreign Secretary Shahidul Haque was competing for the position of deputy director general of the IOM but lost the election (Kawser 2019). The statement of a Bangladeshi embassy official showcases how the candidacy was part of Bangladesh’s greater migration governance strategy: “[A]s a source, transit, and destination country of migration and as a strong promoter of regular migration*, Bangladesh always tries to be at the forefront of any migration discourse*. And in that context, our government decided to nominate Mr. Haque as a candidate for the deputy director general position of the IOM.”^4^

Furthermore, the Bangladeshi ambassador to the UN co-facilitated the International Migration Review Forum (SDG Knowledge Hub 2019) in 2022 as a platform to discuss and share progress on the implementation of the GCM (United Nations Network on Migration n.d.a.). This overview of Bangladesh’s activities demonstrates that the country aims to position itself as a leader in the global governance of migration. The CP is thereby one site among others, albeit important as it works as a linkage and a site of norm diffusion between global fora and regional levels of migration governance.

Bangladesh’s successive governments’ recognition of the significance of their migrant population is showcased in the creation of special government bodies and policies for its international migrant workers, focusing on recruitment regulation and welfare/protection. The Ministry of Expatriates’ Welfare and Overseas Employment was established in 2001 to monitor private recruitment agencies and issue clearances (Agunias, Aghazarm, and Battistella 2015). Bangladesh’s commitment to a formal legal framework of migration governance is evident in its ratification of the Convention on the Protection of the Rights of All Migrant Workers and Members of Their Families (2001).

The 2013 OEMA and the Expatriates’ Welfare and Overseas Employment Policy 2016 are the key policies guiding Bangladesh’s emigration governance (see IOM Bangladesh 2018b). Both acts institutionalize Bangladeshi emigrant rights. All Bangladeshi migrants, including domestic workers, are attributed basic labor rights under the former act, which stipulates that they have the right to legal remedy, including access to labor courts, other courts, and labor attachés in destination countries overseeing their rights (see Overseas Employment and Migrants Act 2013). Again, these policies and strategies are not only an end to themselves but also contribute to building a “brand” Bangladesh by demonstrating being a model state regarding a rights-based migration governance framework, including policy development and implementation, similar to the Philippines.

In 2020, Bangladesh presented the “Bangladesh Migration Governance Framework” (MiGOF) as a guiding framework “for facilitating better coordination among relevant partners and creating a more effective migration governance environment (…)” (Barkat, Osman, and Ahmed 2020, iv). Bangladesh’s MiGOF results from an IOM initiative, where IOM member states internally conceptualized a framework that defines “the elements of well-managed migration or good governance of human mobility” (Barkat, Osman, and Ahmed 2020, vi). In this context, Bangladesh reaffirms its claims to a normative leadership role, emphasizing its status as a “pioneer in developing a national MiGOF” (Barkat, Osman, and Ahmed 2020, iii). It underlines this commitment by pointing to the direct alignment of this framework with the Sustainable Development Goals (SDGs), the Global Compact for Migration, and the national development plan.

An important aspect of branding migration from Bangladesh is to build a reputation of its migrants as regular as opposed to undocumented ones:

Our aim or purpose is to streamline regular migration and stop irregular migration at any cost. Probably in some recent newspaper, [it reported that] there were a few Bangladeshi nationals who were going from Tunisia to Italy. They were apprehended and many of them died in the Mediterranean Sea. This gives a bad name to Bangladesh internationally. But we are one of the top three fastest-growing economies in the world right at this moment, having over 8% GDP growth. We are expecting to have double-digit economic growth by 2024. And hopefully, we will be able to sustain this economic growth for the next 15, 20 years. And we would like to be a developed country by 2041. This is the policy decision of our government, and we are working for that. So, we don’t feel that our workers or our people will die in the Mediterranean Sea.^5^ (Embassy Official)

To compete for international labor market placement and to increase the returns of labor migration, Bangladesh also prioritizes skills policy and schemes. According to the IOM Bangladesh (2018a), “[e]stimates by recruitment agencies suggest that for every $100 earned by a Bangladeshi, $200 is earned by an Indian worker and $300 by a Sri Lankan”. Skill policies form a relevant part of Bangladesh’s strategy to address the current and future development goals and targets of the country which are closely tied to international migration as described earlier. In 2011, Bangladesh adopted a National Skill Development Policy and with the support of IOs, such as the ILO and IOM, has completed several projects and studies aiming to create a framework for enhancing migrant workers’ skills and their recognition.

### India

India has developed into the largest emigrant-origin state globally. In 2024, according to the official numbers of the Indian Ministry of External Affairs, approximately 15.8 million Indians were living abroad (MEA 2024). From the overall population, an estimated 1.1% migrate (The World Bank 2019). About half of all Indian migrants – an estimated 7.9 million – live in the GCC countries (MEA 2024b). About seventy percent of Indian nationals in the GCC countries work in low-wage employment (Khadria 2006). In 2022, remittances represented 3.3 per cent of the country’s GDP (The World Bank Group 2024). Importantly, the significance of emigration to the GCC area varies across different Indian states (Burmeister-Rudolph 2023a).

India has shown low involvement in the CP compared to other participating states. After 21 years of the inception of the CP, it took up its first chairship in 2024 as part of the rotating system^6^ and has not led any TAWGs. Although state ministers have occasionally attended senior official meetings, such as in Colombo (2003) and Dhaka (2011),^7^ India’s commitment is still lower than that of Bangladesh, Nepal, and Sri Lanka, which regularly send ministers to these meetings.

As I will show in the following paragraphs, the Indian state has explicitly created the category “low-skilled emigrant”, and reproduces this category, resulting in acts of omission. According to Menjívar (2023, 12), “when the state omits and neglects it draws a line that excludes, defining an ‘anti-category.’ This category includes those who are ignored, underestimated, and neglected”. Omission is reflected in the exclusion of low-wage migrants in India’s symbolic and substantive emigrant policies and in India’s reluctance to take up more responsibilities within regional and global fora of migration governance. Although India’s emigration policy towards low-wage emigrants is highly regulative, I understand India’s acts of omission in other policy fields, related to migration, such as RCPs like the CP, as examples of strategic non-regulation, a generic form of governing migration (Natter, Norman, and Stel 2023). To make the absences and ambivalences visible, I contrast the Indian state’s actions towards what it categorizes as low-skilled emigrants with those aimed at high-skilled emigrants.

India’s act of indifference, omission, and ambivalence towards low-wage emigrants, apparent in its positioning within the CP, various other multilateral fora, and internal policies, stand in connection to the country’s specific situatedness in the politics of skill, where persons considered as working in low-skilled occupations have been historically devalued and stigmatized. Human development indicators continue to be lowest for Scheduled Castes, Scheduled Tribes, and Muslims; its inter-group distribution reflects social and cultural inequalities (Jayal 2009). These groups also often work in the informal economy in occupations labeled as low-skilled, inflected by caste, religion, and gender inequalities (Jayal 2009). However, scholarship that explores the religious or caste composition of Indian emigrants quantitatively or qualitatively is extremely limited (see Kumar and Rajan 2014), except for the case of the South Indian state of Kerala (Kumar and Rajan 2014). There exists no official statistical data on emigrants concerning these classifications by the Indian state, which can be read as an act of omission.

The independent Indian state has considered ECR-classified emigrants to be legatees of indentured migrants and, as such, undesirable representatives of India overseas (Natarajan 2022; Thakur 2023). Until 1967, the Indian state used a discriminatory passport policy to limit and prevent the emigration of “lower” caste and class citizens, labeled as unskilled and associated with indentured labor migrants, ensuring they were contained within India’s territorial limits (Natarajan 2022). This system upheld India’s international reputation and status, reinforcing the mutually constitutive nature of domestic and foreign citizenship, allowing only ideal, representable migrants – upper caste and class Indians – to represent India internationally. Thakur (2023, 137) points out, “[D]iplomacy (…) becomes a practice through which this difference between the upper caste Indian migrant as a rights-bearing individual and the lower caste Indian migrant as a non-rights-bearing individual is enacted. (…) [C]aste becomes the condition of possibility of India’s diplomacy, of who is worth representing and who is not”. I subsequently demonstrate that this holds also for the arena of migration diplomacy, such as the CP and other multilateral and domestic policies directed at emigrants.

India has no emigrant policy beyond the 1983 Emigration Act. It has provided transnational social protection schemes for low-wage labor, female, and student migrants since 2003 (MEA 2021a). These schemes are unanchored in law, so they can be easily revoked. Social insurance is mandatory for ECR-categorized emigrants, but these schemes mostly provide means-tested social assistance, with Indian missions allocating funds (MEA 2023).

Despite the enfranchisement of Indian citizens residing abroad in 2010, the requirement to cast votes in person in the voter’s constituency often leads to the de facto disenfranchisement of international low-wage migrants (see Burmeister-Rudolph 2023b). Indian migrants’ participation in their home country elections depends on their political importance to parties, which varies by region and influences the arrangement of flights for voting (Burmeister-Rudolph 2023b). The absence of political accountability reduces the incentives for national political parties to address the concerns of low-wage migrant workers. This includes a reluctance to reform the 1983 Emigration Act (see Burmeister-Rudolph 2024).

India, like Bangladesh, acknowledges migrants’ contributions to socio-economic development, but they are not central to its development strategy. Importantly, it credits contributions mainly to high-skilled Indian emigrants – persons residing in countries of the Global North (see Agarwala 2022; Burmeister-Rudolph 2023b), despite over half of India’s remittances coming from the GCC region (RBI Bulletin 2018). Their contributions are discursively omitted from public debates surrounding the migration and development nexus.

India’s choice to endorse the GMC was unexpected, considering its longstanding hesitance to commit to international migration agreements (Akhil 2019). Its plenary statement in the 2022 International Migration Review Meeting is illustrative of India’s acts of omission towards low-wage emigrants, and its incoherent and contradictory policymaking regarding migration governance more generally in three ways. First, it stresses the importance of skilled migration and describes the action the state has taken to achieve its policy goal: “The key pillars of our migration policy are safe, secure, and skilled migration. The Government set up a national ministry in 2014 which is tasked with streamlining vocational and technical training. The focus is on skill up-gradation compliant with new-age courses such as data analysis, smart agriculture, cloud computing, etc.” (Muraleedharan, Minister of State, Government of India 2022).

The statement shows that low-wage migrants are not part of India’s national priorities concerning migration, as they do not fit the description of skilled migrants. In a practical sense, the courses outlined target potential migrants who are already semi-skilled as these would require at least some technical and analytical skills. Moreover, the priority of facilitating more skilled migration through skill enhancement seems to be more of a vision than actual policy: migration does not feature as a component of India’s 2011 National Policy for Skill Development and Entrepreneurship (Ministry of Skill Development and Entrepreneurship 2020). Furthermore, the Pravasi Kaushal Vikas Yojana, a skill development initiative by the Ministry of External Affairs, seeks to elevate the skill levels of prospective emigrant workers in specific sectors and, to align with international standards to promote opportunities for overseas employment, particularly in the GCC region, has been limited to a one-day pre-departure training focusing on soft skills, although it was initially designed to consist of a 160-hours long technical and soft skill training (Ministry of Skill Development and Entrepreneurship 2019; 2023).

Second, the statement stresses the importance of multilateralism and draws a connection to the CP. However, the examples named thereafter are all bilateral agreements with non-GCC countries, namely countries of the Global North:

7. Migration can only be managed collectively. Multilateral understanding is necessary and bilateral partnerships are essential. The Global Compact on Migration and regional groupings such as the Abu Dhabi Dialogue and Colombo Process play an important role, not only to disseminate awareness but also in identifying and sharing of best practices.

8. For instance, India-EU Common Agenda on Migration and Mobility led to agreements with several EU countries, including France (2018), Portugal (2021). An agreement with Germany has recently been finalized. We have signed similar mobility agreements with United Kingdom (2021) and MoC on Specified Skilled Workers with Japan (2021) outside the EU.^8^

Third, the statement highlights the achievements regarding migration agreements with European countries, but obscures India’s inactiveness regarding the implementation of best practices as the CP promotes, for example, the fostering of ethical recruitment practices by reforming its emigration law. To put this into context, India signed 14 Migration and Mobility Partnership Agreements (MMPA) and Declarations of Intent on Migration and Mobility between January 2015 and March 2023 with Global North countries (Singha 2023). It also has Memoranda of Understanding (MOUs) with several GCC countries. The difference in these agreements is that MMPAs cover migrants’ social rights like social security transfers and family reunification. Moreover, MOUs are not enforceable by law. This means that even if they mention social security benefits, they do not guarantee the same legal right to those benefits as a formal, legally binding agreement would (ILO 2023).

## Conclusion

This contribution investigated migrant-origin states as central actors in the politics of skill, more specifically, in the reproduction and reformulation of the category of skill concerning social classifications of class and caste. I juxtaposed and analyzed the actions and behavior of India and Bangladesh in the policymaking towards their low-wage employed emigrants, referring to the Colombo Process as a point of departure to study multiple sites of migration governance where the politics of skill become apparent. India and Bangladesh demonstrate almost opposite behavior when it comes to their current stance regarding low-wage employed emigrants despite sharing similarities. Both countries are significant origin-states of low-wage migrants who predominantly work in the GCC region. Importantly, they inherited the British Indian emigration governance system which differentiated among emigrants based on categories of skill, which are inherently attached to social classifications of caste and class. India continues to structure its policymaking around the two-category distinction, while Bangladesh does not differentiate between its emigrants.

Using the analytical framework of the social construction of skill, I investigated the meaning and therefore value attached to the category of “low-skill” to understand why and how states’ categorization systems of labor emigrants change or remain. This article showed that the two countries’ conduct in multilateral fora of migration governance and domestic policies are linked to the category of skill, and migration governance becomes a site where the category of skill is negotiated, reproduced, and reformulated. In the context of transnational labor migration, the state category of skill often becomes a social category, conflated with classifications such as class and nationality, and a marker of difference.

In the case of Bangladesh, the state recognizes the contributions and value of its emigrant workers and centers its development strategy around them, and accordingly has developed a comprehensive set of migration governance infrastructure domestically and internationally. Categories, such as skill or legality (see Gezahegne Wotere 2024), are not only domestically negotiated but also defined by the international. Therefore, Bangladesh aims to “re-brand” its emigrants – de-linking Bangladeshi nationality from being low-skilled – by promoting itself as a leader regarding the design, promotion, and implementation of global standards of migration governance to increase its market share. The CP provides an ideal platform for Bangladesh’s endeavor with its technical and knowledge support as well as higher bargaining power in coming together with like-minded states.

India, on the other hand, does not challenge the consequences of the social classification of its low-wage employed migrant workers in the GCC country. As this article shows, similar to the destination context, in India “low-skill” has become normalized as a social category, which becomes re-constitutive through the state’s non-policies and acts of omission and reinforces broader societal inequalities and discrimination. The de facto disenfranchisement of Indian emigrants, the regional disparities in emigration rates, remittances, and the political significance of emigrants resulting in their lack of political representation at the national level, are factors that collectively reduce the impetus for reforming the 1983 Emigration Act, thereby perpetuating colonial categorization practices.

This research does not assess the quantifiable impact of these efforts on wage negotiation and better wages for Bangladeshi migrants in the GCC region; however, given that current forms of labor migration are embedded in systems of capital accumulation that run on the racialized hierarchization of (migrant) bodies, it is questionable if Bangladesh can succeed in this endeavor unless workers of other nationalities with even less racial capital (see Alloul 2020) take the place of Bangladeshis. Nevertheless, from a domestic perspective, Bangladesh effectively provides a more comprehensive institutional and policy framework compared to India due to the way the state links the category of “low-skill” with a positive impact. This means, at least on paper, that the rule of law for labor emigrants’ rights can be mobilized and the Bangladeshi state can be held accountable, whereas the absence of specific labor laws for Indian emigrants means legal claims-making for them is inherently limited.

This article has limited itself to studying state practices of constructing categories of skill, focusing on how constructions of skill intersect with class and caste. Given the extreme consequential effects of state categories on individual migrants (Bialas, Lukate, and Vertovec 2024), an obvious direction for future studies is to examine the contestation and negotiation of Indian emigration categories by migrants through their practices. Lastly, this study has not specifically included a discussion of how migrant origin states’ policies towards migrants which are categorized as low-skilled are deeply gendered, but recognizes this dynamic. Scholars (e.g. Shivakoti, Henderson, and Withers 2021) have pointed out how many leading migrant-origin states, including Bangladesh and the Philippines, have been deploying so-called migration bans for domestic workers, who are predominantly female. At first sight, contrary to an active labor “export” strategy and often anchored in paternalistic discourses of protection, bans also function to negotiate better wages (Burmeister-Rudolph 2023b). Moreover, India limits the emigration of ECR-categorized female migrants to an age minimum of 30 years (Agarwala 2022). Bangladesh has imposed an age minimum of 25 years since 2003, while in the previous years, it did not allow any woman categorized as low-skilled (mostly domestic workers) to emigrate (Bélanger and Rahman 2013), which contradicts the country’s attempts to be a norm leader in migration governance, given that the promotion of equality for women migrant workers is one of the cross-cutting themes of the CP. It has yet to be seen if Bangladesh’s ratification of the Domestic Workers Convention in 2023 and thereby the recognition of domestic work as a profession will also lead to a re-valuing of its international domestic workers.

## Data Availability

Due to the sensitive nature of the research interview transcripts are not available.

## Notes

1 Because of social distancing measures, limits to international traveling due to the Covid-19 pandemic and the limited duration of the project through which this research has been financed, traveling to, and conducting interviews in-person in Bangladesh was not possible.

2 The threshold was later lowered to ten years of schooling, see Introduction of this article.

3 There are 33 champion countries in total: Cambodia, Indonesia, Nepal, Philippines, Thailand are the other CP states (United Nations Network on Migration n.d.b.).

4 Interview, Bangladesh High Commission, 19 March 2019, Den Hague.

5 Interview, Bangladesh High Commission, 19 March 2019, Den Hague.

6 This article was written in 2023 and revised in May 2024, so it does not consider India's chairship in its analysis.

7 H.E. Dr. Sahib Singh, Minister of Labor (2003), H.E. Mr. Vayalar Ravi, Minster of Overseas Indian Affairs (2011) (see Colombo Process 2003a, 2011a).

8 Muraleedharan, Indian Minister of State 2022.
